# Tenosynovial giant cell tumor: a case report

**DOI:** 10.1186/s13256-023-04156-w

**Published:** 2023-10-06

**Authors:** Sonam Ansel, Xiangfei Yan, Peter Chong, Steven Lo, Mark McCleery, Ashish Mahendra, Elaine MacDuff, Fiona Cowie, Ioanna Nixon, Jeff White, the Scottish Sarcoma Network

**Affiliations:** 1 Beatson West of Scotland Cancer Centre, Glasgow Royal Infirmary, Glasgow, Scotland; 2 Departments of General Surgery, Glasgow Royal Infirmary, Glasgow, Scotland; 3 Departments of Plastic Surgery, Glasgow Royal Infirmary, Glasgow, Scotland; 4 Departments of Radiology, Glasgow Royal Infirmary Glasgow Royal Infirmary, Glasgow, Scotland; 5 Departments of Orthopaedic Oncology, Glasgow Royal Infirmary, Glasgow, Scotland; 6 Department of Pathology, Queen Elizabeth University Hospital, Glasgow, Scotland

**Keywords:** Tenosynovial giant cell tumor, Sarcoma, Pigmented villonodular synovitis, Joint tumors

## Abstract

**Background:**

This case reports the synchronous diagnosis of two rare unrelated diseases; leiomyosarcoma and tenosynovial giant cell tumor of the knee. It focuses on the challenges of diagnosing tenosynovial giant cell tumor, including cognitive biases in clinical medicine that delay diagnosis. It also demonstrates the pathogenic etiology of tenosynovial giant cell tumor, evidenced by the transient deterioration of the patients’ knee symptoms following the administration of prophylactic granulocyte colony-stimulating factor given as part of the chemotherapeutic regime for leiomyosarcoma.

**Case presentation:**

A 37-year-old Caucasian man presented with a left groin lump and left knee pain with swelling and locking. Investigations including positron emission tomography-computed tomography and biopsy revealed leiomyosarcoma in a lymph node likely related to the spermatic cord, with high-grade uptake in the left knee that was presumed to be the primary site. His knee symptoms temporarily worsened each time granulocyte colony-stimulating factor was administered with each cycle of chemotherapy for leiomyosarcoma to help combat myelosuppressive toxicity. Subsequent magnetic resonance imaging and biopsy of the knee confirmed a tenosynovial giant cell tumor. His knee symptoms relating to the tenosynovial giant cell tumor improved following the completion of his leiomyosarcoma treatment.

**Conclusions:**

Tenosynovial giant cell tumor remains a diagnostic challenge. We discuss the key clinical features and investigations that aid prompt diagnosis. The National Comprehensive Cancer Network clinical practice guidelines for soft tissue sarcoma have recently been updated to include the pharmacological management of tenosynovial giant cell tumor. Our case discussion provides an up-to-date review of the evidence for optimal management of patients with tenosynovial giant cell tumor, with a particular focus on novel pharmacological options that exploit underlying pathogenesis.

## Background

As per the 2020 World Health Organization (WHO) classification, tenosynovial giant cell tumor (TGCT) encompasses a group of lesions most often arising from the synovium of joints, bursae, and tendon sheaths and showing synovial differentiation [1]. The vast majority are benign but locally aggressive tumors. TGCTs are usually divided according to their growth pattern (diffuse or localized) and site (intra- or extraarticular). Formerly known as pigmented villonodular synovitis (PVNS), diffuse TGCTs are rare, with only four cases per million person-years [1]. It generally presents in patients aged 20–40 years and there is a slight female predominance [1]. The etiology is related to overexpression of colony stimulation factor 1 (CSF-1), which is a hemopoietic growth factor and results in soft tissue hyperplasia in synovial cells lining joints. Most patients with diffuse TGCT report swelling, tenderness, pain, or limitation of motion, whereas patients with the localized type more commonly report a painless mass. Onset of symptoms can be initially insidious, however, they can become debilitating if left untreated. The complications of diffuse TGCT include joint deformity, degenerative articular changes, and osteoarthritis.

TGCT remains a diagnostic challenge. Due to its slow onset, non-specific symptoms, and subtle radiographic changes, the median time from the onset of symptoms to definitive diagnosis has been shown to be 18 months [2]. This can be a frustrating and unsatisfactory period for both the patient and physician. This case aims to highlight common diagnostic pitfalls, with focus on the CSF-1-related pathogenesis reflected by a worsening of symptoms with granulocyte colony-stimulating factor (GCSF). Surgical resection forms the mainstay of treatment for patients with TGCT. However, novel drugs targeting the CSF-1 pathway have been explored, and pexidartinib, a CSF-1 receptor antagonist, was approved by the US Food and Drug Administration (FDA) in 2019 for patients with extensive disease not suitable for surgical intervention [3].

## Case presentation

A 37-year old Caucasian man presented to the emergency department with a 6-week history of a painless, left inguinal lump. There were no systemic symptoms. He had no significant past medical history, was a non-smoker, did not drink alcohol, and was not on any regular medication. He worked as a self-employed joiner. Family history included leukemia in a paternal uncle, pharyngeal cancer in a paternal aunt, and Ewing’s sarcoma in a maternal aunt.

**Fig. 1:**
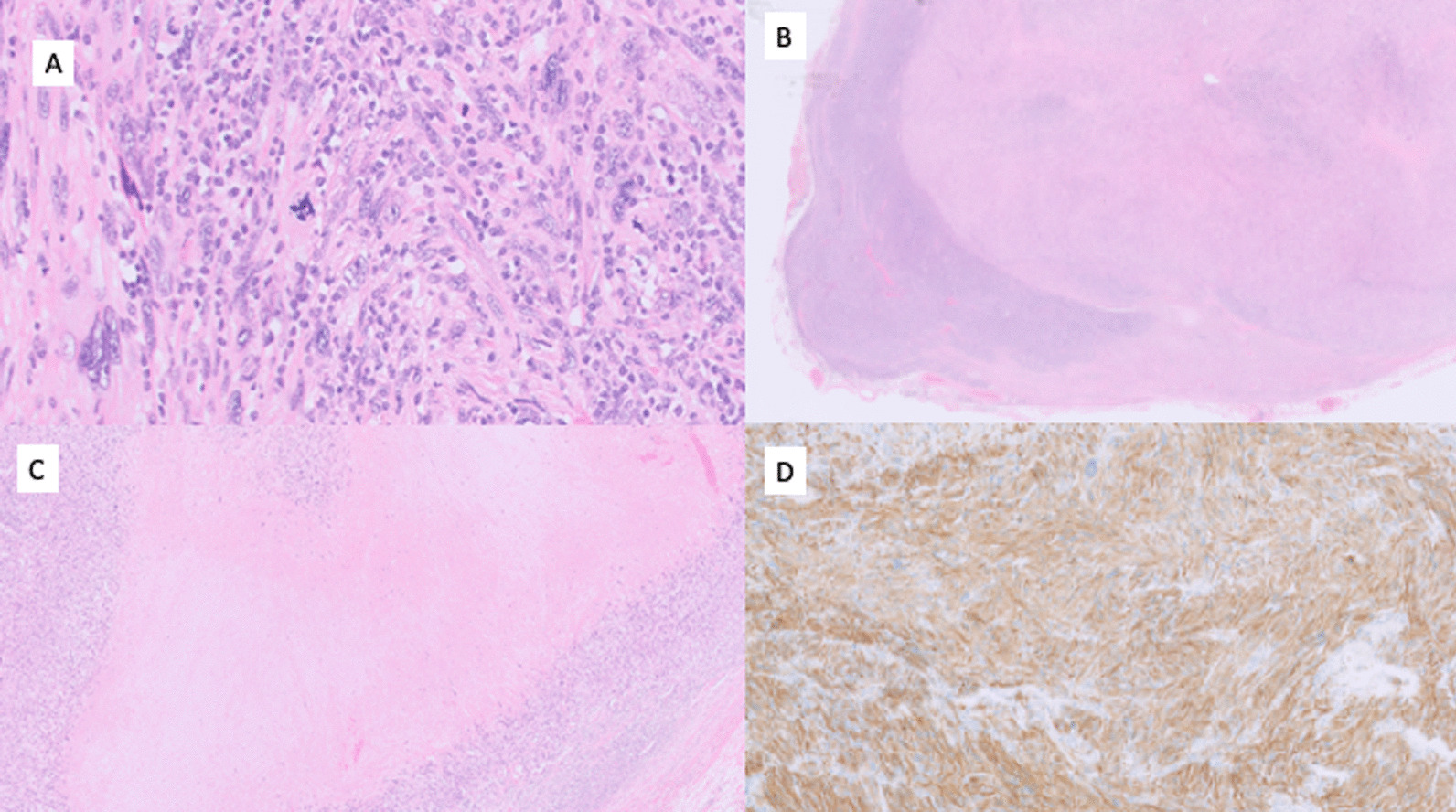
Microscopy images of intranodal grade 3 leiomyosarcoma. **A** High power, demonstrating atypical mitosis and nuclear atypia. **B** Low power, demonstrating tumor within lymph node. **C** Medium power, demonstrating central areas of necrosis. **D** Medium power, demonstrating positive desmin staining

On examination, there was a well-circumscribed, irreducible, and nontender lump above the pubic tubercle. An ultrasound was performed and it reported appearances suspicious of an abnormal lymph node. A biopsy was performed and pathology was in keeping with a Trojani grade 3 intranodal leiomyosarcoma; therefore, it was considered a nodal metastasis. This tumor demonstrated positivity to desmin, muscle specific actin (MSA), smooth muscle myosin (SMM), and caldesmon, but was negative for S100, CD34, and CD31. There was necrosis, with mitotic figures identified with 25 mitoses per 10 high power fields including numerous atypical forms (Fig. 1). The surgeon also commented that the lymph node was unusually deep, with close relation to the spermatic cord.

**Fig. 2:**
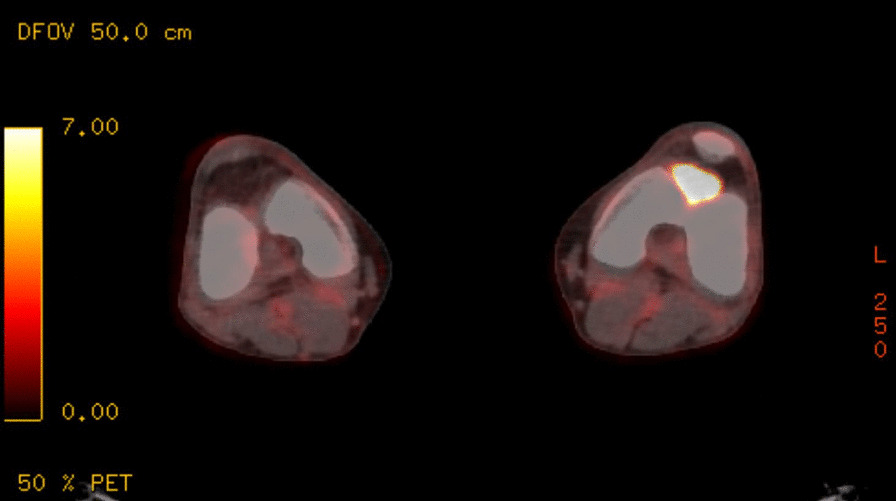
Positron emission tomography-computed tomography image of knees showing a fluorodeoxyglucose-avid mass within the left knee, lying immediately anterior to the intercondylar groove and posterior to the patella

Computed tomography (CT) chest, abdomen, and pelvis showed no other sites of disease. However, positron emission tomography (PET) revealed a fluorodeoxyglucose (FDG)-avid mass within the left knee (Fig. 2), as well as nodal activity in left groin and left external iliac region. On the basis of the PET scan findings, the knee mass was assumed to be the primary site for the leiomyosarcoma, with metastases to the left groin node. Magnetic resonance imaging (MRI) of the left knee was also performed, but was unreported at the time of commencing neoadjuvant chemotherapy. The patient proceeded to receive neoadjuvant chemotherapy, with four cycles of doxorubicin and ifosfamide chemotherapy for treatment of metastatic leiomyosarcoma. Prophylactic GCSF subcutaneous injections is the standard given, following each cycle to combat neutropenia, a common hematological toxicity [4].

The patients’ knee symptoms deteriorated while receiving treatment for the leiomyosarcoma. A cyclical relationship between the knee pain and administration of GCSF following chemotherapy was noted, and at it’s worst, he was unable to bear weight and a large, tender effusion developed.

**Fig. 3:**
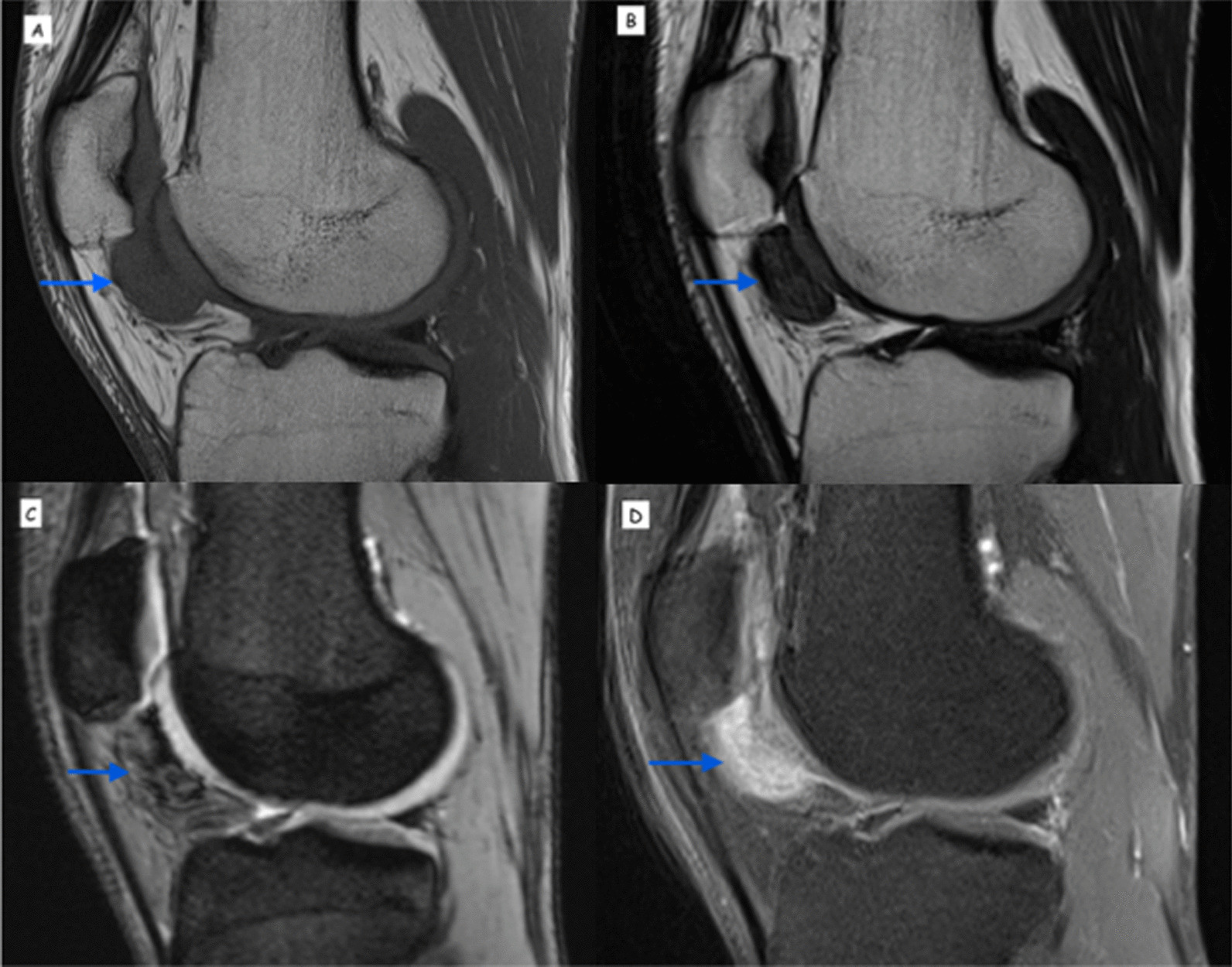
Sagittal magnetic resonance imaging of the left knee demonstrating the well-defined, predominantly low-signal, diffuse tenosynovial giant cell tumor lesion in the inferomedial gutter of the patellofemoral joint (arrows point to the diffuse tenosynovial giant cell tumour lesion). **A** T1-weighted image **B** T2-weighted image **C** T2 gradient echo sequence (GRE) showing bloom artifact **D** T1 weighted fat-saturated image

MRI knee was carried out several weeks into his chemotherapy, which noted a well-defined soft tissue lesion demonstrating features in suggestive of diffuse TGCT (Fig. 3). The patient continued with chemotherapy under the clinical presumption that this was primary leiomyosarcoma with inguinal nodal metastasis, and an interval MRI in 3 months was planned to assess response to chemotherapy.

**Fig. 4:**
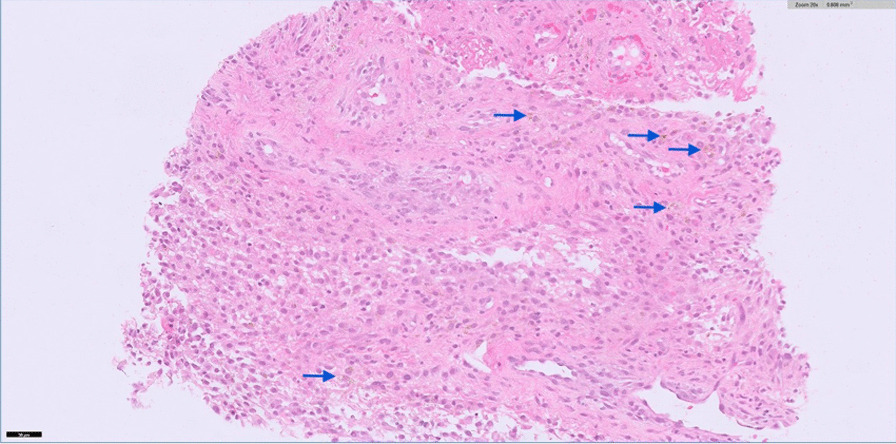
Microscopy image of synovium biopsy core (zoom 0.5×). Fibrous tissue and sheets of mononuclear histiocytes. Intra- and extracellular deposition of hemosiderin pigment (arrows point to hemosiderin pigment). There are no giant cells, but overall the material is consistent with diffuse tenosynovial giant cell tumour

Repeat MRI knee at the end of neoadjuvant chemotherapy demonstrated similar features suspicious of TGCT, and this was subsequently confirmed following biopsy of the synovium (Fig. 4).

Following the completion of chemotherapy, the patient underwent surgery (wide local excision of the left groin lesion, left orchidectomy, resection of spermatic cord, and groin flap reconstruction). No residual tumor was seen on pathology. At the time of writing this case report, the patient is 42 months into his follow-up with no signs of recurrence. The patient continues to complain of left knee pain and locking, however, to a much lesser degree and it does not interfere with his activities of daily living.

## Discussion

### Clinical evaluation

TGCT is challenging to diagnose due to the relatively non-specific signs or symptoms at presentation, which cover wide differential diagnoses [5, 6]. Plain X-rays may be unremarkable, and MRI of the affected joint is the most sensitive imaging modality [7–10]. Histopathological analysis is necessary to confirm diagnosis, with microscopy often revealing mononuclear cells (including histiocytes), macrophages with extensive hemosiderin stores, and multinucleated osteoclast-type giant cells [11]. There are diffuse expansive sheets of cells with infiltrative borders and variable cellularity, and tumor margins are more cellular [12]. Giant cells are not observed in about 20% of diffuse TGCT cases, which adds to the diagnostic challenge [9]. Serum inflammatory markers, such as erythrocyte sedimentation rate (ESR) and C-reactive protein (CRP), are not elevated in the majority of patients [13].

### Management options

There is a lack of consensus regarding optimal management of TGCT, but surveillance, conservative management, surgery with synovectomy, and radiotherapy are all options [14]. Total synovectomy may be difficult to achieve in the diffuse subtype, due to the risk of neurovascular damage. Local recurrence rates range from 0% to 15% (localized) to up to 50% (diffuse subtype) [15].

External beam radiotherapy (EBRT) can be used in the postoperative setting after incomplete resection to reduce risk of local recurrence, or as a salvage option for treatment of recurrences. The benefits are difficult to quantify due to lack of evidence, but a meta-analysis in 2015 of 35 observational studies found that the rate of local recurrence of diffuse TGCT in the knee was significantly reduced with perioperative EBRT (from 36.9% to 12.0%) [16]. The literature describes radiation doses of 20–50 Gy in 15–25 fractions, starting within 6–8 weeks of surgery [17].

Radiosynoviorthesis (RSO), which involves the intraarticular injection of a radioisotope, is described in two small retrospective case–control studies. One study concluded that adjuvant RSO may reduce rate of local recurrence in select patients, whereas the other was unable to provide conclusive evidence of any benefits derived from the adjuvant treatment [18, 19].

Recently, improved understanding of the pathology underlying TGCT has exposed a potential role for systemic therapies as part of the overall management of the disease process. TGCT results from overexpression of colony stimulating factor 1 (CSF-1) in synovial cells, and recruitment of CSF1 receptor (CSF-1R) on macrophages, giant cells, and osteoclasts results in hyperplasia. In the majority of cases, there is an underlying chromosomal translocation, t(1;2) (CSF-1;COL6A3) [20]. Although GCSF (also known as CSF-3) acts primarily on neutrophils and neutrophil progenitors, it can also act upon monocytes, which differentiate into macrophages [21], and the binding of GCSF to monocytic cells has been demonstrated in *in vitro* studies [22]. Our case reinforces the latter role, as the patient’s TGCT symptoms worsened following administration of GCSF.

There is ongoing research focusing on systemic therapies targeting the CSF-1 pathway, including monoclonal antibodies and tyrosine kinase inhibitors. Pexidartinib, a CSF-1R antagonist, is the first drug to be approved by the FDA for adults with TGCT associated with severe morbidity or functional limitations without improvement despite surgical intervention. The ENLIVEN study showed a higher objective response rate for pexidartinib compared with the placebo, but highlighted the risk of serious liver injury [3]. The National Comprehensive Cancer Network clinical practice guidelines for soft tissue sarcoma list pexidartinib as a category 1 recommendation [23]. Imatinib is approved for use in management of hematological malignancies, dermatofibrosarcoma protuberans, and gastrointestinal stromal tumors. Its principal mechanism of action involves targeting tumoral BCR–ABL1 and KIT oncogene products [24]. However, there is growing evidence that indicates further therapeutic uses of imatinib due to its modulatory effects on protein tyrosine kinases involved in key signalling pathways implicated in cancer surveillance [24]. An international multi-institutional retrospective study in 2019 confirmed efficacy of imatinib in patients with locally advanced, recurrent, or metastatic diffuse TGCT [25].

## Conclusions

This case has presented an extremely unusual clinical picture and the challenges involved in diagnosis, in which a patient was diagnosed with synchronous rare conditions: TGCT of the knee and metastatic leiomyosarcoma within an inguinal lymph node without an obvious primary source. It further highlights the pathology of TGCT through the transient worsening of symptoms with GCSF and demonstrates that a multidisciplinary approach is key to improving outcomes for patients with TGCT.

## Data Availability

Not applicable.

## Abbreviations

TGCT: Tenosynovitis giant cell tumor
PVNS: Pigmented villonodular synovitis
NCCN: National Comprehensive Cancer Network
GCSF: Granulocyte colony-stimulating factor
WHO: World Health Organization
CSF-1: Colony stimulating factor 1
ESR: Erythrocyte sedimentation rate
CRP: C-reactive protein
EBRT: External beam radiotherapy
RSO: Radiosynoviorthesis
